# Possible role for nephron‐derived angiotensinogen in angiotensin‐II dependent hypertension

**DOI:** 10.14814/phy2.12675

**Published:** 2016-01-12

**Authors:** Nirupama Ramkumar, Deborah Stuart, Matias Calquin, Shuping Wang, Fumio Niimura, Taiji Matsusaka, Donald E. Kohan

**Affiliations:** ^1^ Division of Nephrology and HypertensionUniversity of Utah Health Sciences CenterSalt Lake CityUtah; ^2^ Institute of Medical ScienceTokai UniversityIseharaJapan; ^3^ Veterans Affairs Medical CenterSalt Lake CityUtah

**Keywords:** Angiotensinogen, blood pressure, kidney, liver

## Abstract

The role of intranephron angiotensinogen (AGT) in blood pressure (BP) regulation is not fully understood. Previous studies showed that proximal tubule‐specific overexpression of AGT increases BP, whereas proximal tubule‐specific deletion of AGT did not alter BP. The latter study may not have completely eliminated nephron AGT production; in addition, BP was only assessed on a normal salt diet. To evaluate this issue in greater detail, we developed mice with inducible nephron‐wide AGT deletion. Mice were generated which were hemizygous for the Pax8‐rtTA and LC‐1 transgenes and homozygous for loxP‐flanked AGT alleles to achieve nephron‐wide AGT disruption after doxycycline induction. Compared to controls, AGT knockout (KO) mice demonstrated markedly reduced renal AGT immunostaining, mRNA, and protein levels; unexpectedly AGT KO mice had reduced AGT mRNA levels in the liver along with 50% reduction in plasma AGT levels. BP was significantly lower in the AGT KO mice compared to controls fed a normal, low, or high Na^+^ intake, with the highest BP reduction on a low Na^+^ diet. Regardless of Na^+^ intake, AGT KO mice had higher plasma renin concentration (PRC) and markedly reduced urinary AGT levels compared to controls. Following angiotensin‐II (Ang‐II) infusion, AGT KO mice demonstrated an attenuated hypertensive response despite similar suppression of PRC in the two groups. Taken together, these data suggest that nephron‐derived AGT may be involved in Ang‐II‐dependent hypertension, however, a clear role for nephron‐derived AGT in physiological BP regulation remains to be determined.

## Introduction

The intranephron renin–angiotensin system (RAS) may be important as it contains all the elements necessary to generate angiotensin‐II (Ang‐II) (Rohrwasser et al. 1999; Navar et al. 2011; Carey 2015). In this system, angiotensinogen (AGT) is synthesized in the proximal tubule (PT) and secreted into the tubular lumen (Niimura et al. 1997; Ingelfinger et al. 1999; Rohrwasser et al. 1999; Kobori et al. 2002; Lantelme et al. 2002) and renin is produced by the distal nephron and secreted into the tubular lumen (Rohrwasser et al. 1999; Liu et al. 2011). Furthermore, angiotensin converting enzyme is found in abundance throughout the apical nephron surface. As Ang‐II receptors are found throughout the nephron (Oliverio et al. 1998a) and their activation has been shown to stimulate Na^+^ and water reabsorption (Oliverio et al. 1998b; Peti‐Peterdi et al. 2002; Stegbauer et al. 2011; Mamenko et al. 2012), tubular Ang‐II may modulate salt and water excretion and ultimately BP.

Previous studies suggest that the PT is the main site of nephron AGT synthesis (Ingelfinger et al. 1990, 1999; Niimura et al. 1997; Rohrwasser et al. 1999; Pohl et al. 2010). These findings are supported by the observation that mice with human AGT overexpression under the control of the human AGT promoter express high levels of renal AGT selectively in the PT (Ding et al. 1997; Ding and Sigmund 2001). AGT produced by the PT is secreted, at least partly, into the tubule lumen and appears in the urine (Kobori et al. 2002, 2003). Furthermore, PT AGT synthesis and urinary AGT excretion are increased by Ang‐II (Kobori et al. 2001, 2007; Gonzalez‐Villalobos et al. 2008, 2010). The physiological relevance of PT‐derived AGT in blood pressure (BP) regulation has been explored in a series of studies. Transgenic mice with PT‐specific overexpression of human AGT and human renin are hypertensive despite normal systemic RAS parameters (Lavoie et al. 2004). Similarly, PT‐specific rat AGT overexpression caused hypertension (Sachetelli et al. 2006). However, these models have an overactive intrarenal RAS with renin levels clamped several fold higher than in pathological states. To address whether PT AGT in the presence of normal renin levels can regulate BP, we developed a mouse model with PT selective mouse AGT overexpression (Ying et al. 2012). These mice demonstrated salt‐sensitive hypertension, increased urinary AGT and Ang‐II excretion, and suppressed PRC with high Na^+^ intake suggesting that PT AGT may play a role in BP regulation.

A recent study called into question the significance of PT‐derived AGT (Matsusaka et al. 2012); mice with PT‐specific knockout (KO) of AGT had no detectable change in intrarenal AGT, whereas mice with liver‐specific AGT deletion had a marked reduction in intrarenal AGT (Matsusaka et al. 2012). Of note, the liver‐specific AGT KO mice had no reduction in urinary AGT, whereas the PT‐specific AGT KO mice had reduced (by ~50%) urinary AGT. Furthermore, PT‐specific deletion of AGT did not alter BP, whereas liver‐specific AGT KO caused severe hypotension (Matsusaka et al. 2012). These findings suggest that the bulk of the PT AGT derives from the liver and is responsible for intrarenal Ang‐II generation and BP regulation. However, concerns exist with the PT‐specific AGT KO studies. First, it remains unclear whether PT AGT was completely disrupted since urinary AGT was reduced by only 50% in these mice. In addition, studies were only conducted on a normal salt diet and the effect of raising systemic Ang‐II (which as stated above increases PT AGT) was not evaluated. Consequently, we developed a mouse model with complete disruption of nephron AGT. We describe the effects of nephron AGT deletion on BP and sodium excretion under varying dietary salt conditions and following systemic Ang‐II infusion.

## Methods

### Animal care

All animal studies were conducted with the approval of the University of Utah Animal Care and Use Committee in accordance with the National Institutes of Health Guide for the Care and Use of Laboratory Animals.

### Generation of inducible nephron‐wide AGT KO mice

**Figure 1:**
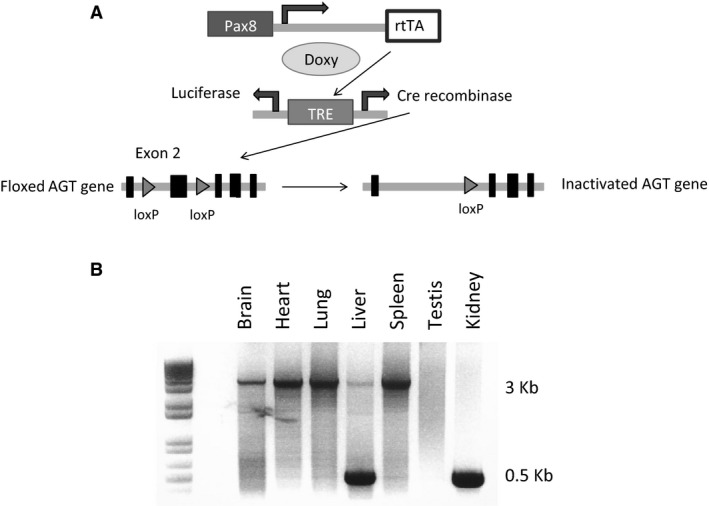
Gene targeting strategy used to generate inducible nephron AGT KO mice and organ‐specific recombination. (A) Pax8 promoter drives expression of the reverse tetracycline transactivator (rtTA), which requires doxycycline to activate the bicistronic Cre recombinase/luciferase expressing transgene. Cre is expressed in the nephron but not in glomeruli. Doxy, doxycycline; TRE, tetracycline response element. (B) Representative blot of AGT gene recombination in various organs in AGT KO mice (*N* = 4 mice). The 3 Kb band is the unrecombined allele and the 500 bp band is the recombined allele.

Details on generation of mice with loxP‐flanked (floxed) exon 2 of the *AGT* gene have been published (Matsusaka et al. 2012). Floxed AGT mice were bred with mice containing Pax8‐rtTA and LC‐1 transgenes. The Pax8‐rtTA transgene contains the *Pax8* gene promoter driving expression of the reverse tetracycline transactivator (rtTA) primarily within the nephron (Traykova‐Brauch et al. 2008). The LC‐1 transgene encodes tetracycline‐inducible bicistronic Cre recombinase and luciferase (Schonig et al. 2002). In the presence of doxycycline, rtTA binds and activates the LC1 transgene leading to expression of luciferase and Cre recombinase in renal tubular cells (Fig. 1A). To induce nephron‐wide knock out, at 1 month of age, mice hemizygous for Pax8‐rtTA, hemizygous for LC‐1, and homozygous for floxed AGT gene were given 2 mg/mL doxycycline in 2% sucrose drinking water for 12 days followed by 4 weeks off doxycycline. Floxed AGT mice without the Pax8‐rtTA or LC‐1 transgenes were used as controls. All mice were bred on a C57BL/6J background, and male floxed and AGT KO mice aged 3–6 months were used for all studies.

### Genotyping

Tail DNA was isolated and PCR performed using the following primers: AGT, forward 5′‐ CATGGTGAGTTCAAGACCAGCTGG ‐3′ and reverse 5′‐ TCCGGGTGGAAAGCACACTCATCC ‐3′, which yields a 240 bp product from the floxed AGT gene and a 200 bp product from the wild‐type allele; Pax8‐rtTA forward 5′‐CCATGTCTAGACTGGACAAGA‐3′ and reverse 5′‐CATCAATGTATCTTATCATGTCTGG ‐3′ which yields a 600 bp product; and LC‐1 forward 5′‐TCGCTGCATTACCGGTCGATGC‐3′ and reverse 5′‐CCATGAGTGAACGAACCTGGTCG‐3′ which yields a 480 bp product.

### Screening for AGT gene recombination

DNA was isolated from a variety of organs and PCR amplified to examine the organ‐specific expression of the recombined targeted *AGT* gene. PCR was performed for 35 cycles at 94°C for 15 sec, 55°C for 30 sec, and 68°C for 4 min using the following primers to amplify the transgene – forward 5′‐GCAGGGCGATTTACTGGACT‐3′ and reverse 5′‐CCTACTGTGGGCTGCGTAAA‐3′. These primers are located in introns 1 and 2 and yield a 3200 bp product in nonrecombined DNA and a 500 bp product in recombined DNA.

### Quantitation of AGT mRNA

Kidneys and liver were dissected from AGT KO and control mice for RNA isolation. Reverse transcription was performed on 2 *μ*g of total RNA with oligo(dt) and Superscript III reverse transcriptase according to the manufacturer's protocol (Invitrogen, Grand Island, NY). The resulting cDNA was then assayed for relative expression of AGT mRNA in KO and floxed mice using a Taqman Gene Expression Assay (AGT probe cat # Mm00599662_m1, GAPDH probe cat# Mm03302249_g1, Applied Biosystems, Carlsbad, CA).

### Immunofluorescence

Kidneys were fixed overnight in 10% formaldehyde, embedded in paraffin and 4 *μ*m sections obtained. Kidney sections were rehydrated with xylene and ethanol and treated with 1% SDS for 10 min to enhance antibody staining. After blocking with 1% BSA in PBS for 1 hr, sections were incubated with primary antibody against AGT (1:25, IBL America, Minneapolis, MN) overnight. After three consecutive washes of 5 min each with PBS, kidney sections were incubated with secondary donkey anti goat Alexa Fluor 488 (1:50) antibodies for 60 min. After three wash‐rinse steps of 5 min each with PBS, slides were mounted in Vectashield (Vector Laboratories, Burlingame, CA) and sealed with a coverslip. Tissue sections were examined and photographed with a Nikon FXA epiflourescence microscope.

### Blood pressure monitoring

Blood pressure (BP) was recorded via telemetry (TA11‐PAC10, Data Sciences International, St. Paul, MN). Control and AGT KO mice were anesthetized with 2% isoflorane, implanted with radio transmitters with the catheter in the carotid artery, and allowed to recover for 5 days. Mice were maintained on a normal Na^+^ diet for 3 days (0.26%), high Na^+^ diet (3.2%) for 6 days and low Na^+^ diet (0.03% Na^+^) for 7 days. Mice were not handled during BP recording period since even small stimuli can markedly affect BP in mice.

### Angiotensin‐II infusion

After 3 days of continuous BP monitoring on a normal Na^+^ diet, mini‐osmotic pumps (Alzet Model 1002, Durect Corporation, Cupertino, CA) were placed subcutaneously in between the scapulae under isoflurane anesthesia. Angiotensin‐II was infused for 14 days at 400 ng/kg/min and BP recorded continuously for 12 days on a normal Na^+^ diet. At the end of the BP studies, mice were placed in metabolic cages for 48 hours for urine and plasma collection. Mice were then sacrificed and kidneys isolated for Na^+^ transporter expression.

### Metabolic balance studies

Control and AGT KO mice were placed in metabolic cages for 18 consecutive days for measurement of food and water intake, body weight and 24‐h urine collection. Mice were given 9 mL of a normal (4 days), high (7 days), or low Na^+^ (7 days) gelled diet with free access to water. On days 4, 11, and 18, 35 *μ*L of blood was collected for assay of plasma renin concentration and plasma AGT. Plasma renin concentration (PRC) was measured as the amount of angiotensin‐1 (Ang‐I) generated after incubation with excess porcine angiotensinogen using the Ang‐I enzyme immunoassay (EIA) kit (Bachem, San Carlos, CA). Plasma and urine total AGT were measured using a commercially available EIA kit (IBL America, Minneapolis, MN). Urinary Na^+^ and K^+^ were determined using the EasyVet Analyzer (Medica, Bedford, MA) and albumin excretion using ELISA kit (Albuwell M kit, Exocell Inc). At the conclusion of metabolic balance studies, mice were sacrificed and kidneys isolated for further analyses.

### Western blotting

A segment of the liver and whole kidneys were isolated and homogenized in ice‐cold sucrose buffer (10 mmol/L triethanolamine, 250 mmol/L sucrose, pH 7.6) with PMSF (100 *μ*g/mL), leupeptin (10 μmol/L) and Complete Protease Inhibitors (Roche, Pleasanton, CA). After centrifuging the samples at 2000 × *g* for 10 min at 4°C, the supernatant was collected and an aliquot was taken for determination of protein content using the Bradford assay (Bio‐Rad, Hercules, CA). The remaining sample was solubilized with Laemmli loading buffer containing 0.5% lithium dodecyl sulfate and heated at 60°C for 10 min. Liver and kidney lysates (10 *μ*g/lane) were run on a denaturing NUPAGE 4–12% Bis‐Tris minigel (Invitrogen), transferred to a polyvinylidene difluoride plus nylon membrane, and visualized with the Advance ECL system (GE Healthcare, Piscataway, NJ). Densitometry was performed with a Bio‐Rad gel documentation system (Hercules, CA). Membranes were incubated with primary antibody against AGT (28101, IBL America, Minneapolis, MN) and after visualization, reprobed with GAPDH antibody (Cell Signaling, Danvers, MA).

For Na^+^ transporter expression within the kidney, membranes were incubated with specific antibodies against NHE3 (1:1000, Millipore, Bedford, MA), NCC (1:1000, gift from Dr. David Ellison, Oregon Health Sciences University) and ENaC‐*α*, (1:1000, StressMarq, Victoria, BC). Secondary horseradish peroxidase‐conjugated antibodies (goat anti‐mouse for NHE3 and goat anti‐rabbit for other transporters, Santa Cruz Biotech, Santa Cruz, CA) were used at a dilution of 1:5000. Densitometry was performed with a Bio‐Rad gel documentation system (Hercules, CA).

### Statistical analysis

All results are expressed as mean ± SEM. The Student's unpaired *t*‐test was used to compare differences between KO and control animals. For parameters requiring more than one comparison, ANOVA with Scheffe post hoc test was used, as indicated. The criterion for significance was *P* ≤ 0.05.

## Results

### Verification of nephron‐wide KO of AGT

PCR of DNA isolated from various organs from nephron AGT KO mouse demonstrated recombination in the liver and kidneys (Fig. 1B). Previous studies have demonstrated that the Pax8‐rtTA/LC‐1 system is highly specific within the kidney for renal tubular epithelial cells, does not target glomeruli (Faresse et al. 2012; Stuart et al. 2012; McCormick et al. 2014), but can target some periportal hepatocytes.

**Figure 2:**
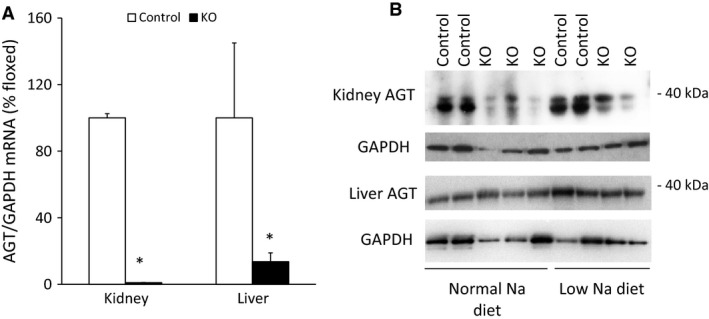
AGT mRNA and protein expression in control and AGT KO mice. (A) AGT mRNA expression by RT‐PCR in liver and kidney (*N* = 6–8/group), values are adjusted to control levels (100%); (B) AGT protein by Western blot in kidney and liver (*N *= 3/group). *P < 0.05 versus control mice.

**Figure 3:**
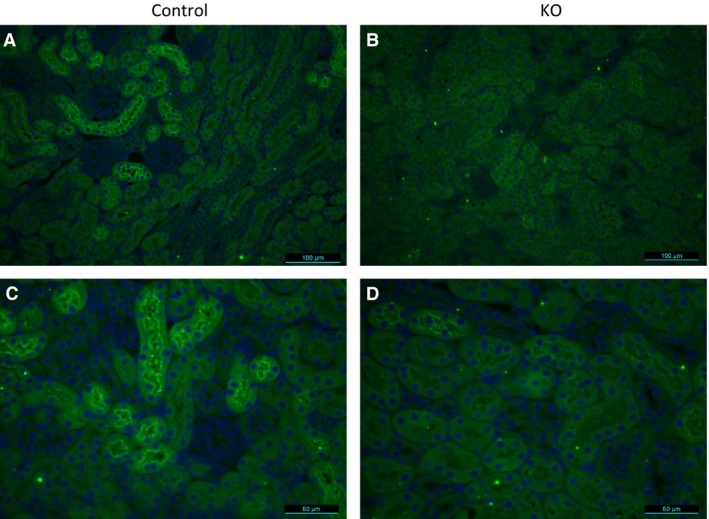
Immunostaining of kidney sections with anti‐AGT antibody (green) from control (A and C) and PRR KO mice (B and D). Images are representative of three different mice. (A and B) are 200× and (C and D) are 400× magnification.

Compared to control mice, renal AGT mRNA and protein levels were markedly reduced in the AGT KO mice (Fig. 2). Hepatic AGT mRNA levels were also reduced in the KO mice, whereas liver AGT protein levels were similar between the two groups (Fig. 2). Renal AGT staining by immunofluorescence was markedly reduced in the AGT KO mice compared to controls (Fig. 3).

### Effect of nephron‐wide AGT KO on blood pressure and Na^+^ excretion

**Figure 4:**
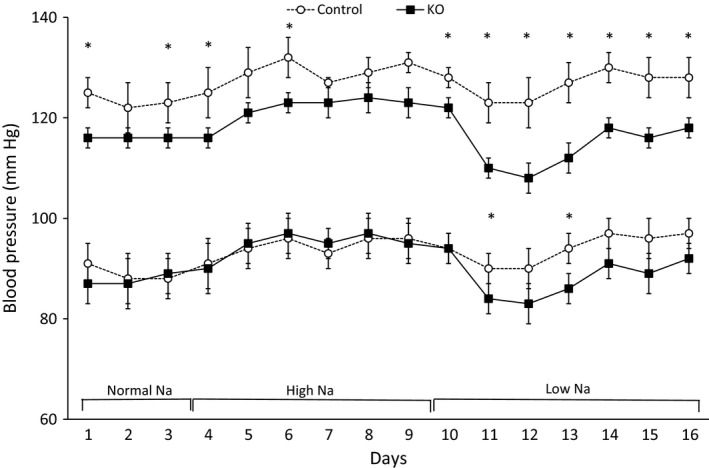
Daily BP in control and AGT KO mice with varying Na^+^ intake. BP was measured via telemetry and mice were fed normal (0.26%), high (3.2%), and low (0.03%) Na^+^ diets (*N* = 8–10/group). Top two lines show systolic, and bottom two lines show diastolic, BP. *P < 0.05 versus control mice.

Compared to controls, AGT KO mice had mildly reduced systolic BP with normal or high Na^+^ intake (Fig. 4). No detectable differences were observed in diastolic BP between the two groups on either normal or high Na^+^ diet. With low Na^+^ intake, AGT KO mice continued to have lower systolic (~10 mmHg) and diastolic (~5 mmHg) BP compared to controls. There were no detectable differences in food intake, water intake, body weight, urine volume or urine Na^+^ excretion between control and AGT KO mice on all three diets (Table 1). Note that the table reports values for Day 3 of each diet, however, no differences were detected in urinary Na^+^ excretion between control and AGT KO mice on other days.

**Table 1:** Food intake, water intake, urine volume, urine Na^+^ excretion, and body weight in control and AGT KO mice, *N* = 8–10 per group

	Normal Na^+^ diet Day 3		Low Na^+^ diet Day 3		High Na^+^ diet Day 3		Post Ang‐II Day 14	
	Control	KO	Control	KO	Control	KO	Control	KO
Food intake (g)	4.7 ± 0.2	4.4 ± 0.2	4.5 ± 0.5	4.2 ± 0.3	4.6 ± 0.3	5.2 ± 0.3	5.4 ± 0.3	4.9 ± 0.1
Water intake (mL)	3.8 ± 0.5	3.8 ± 0.1	3.6 ± 0.5	3.1 ± 0.2	6.1 ± 0.4	6.2 ± 0.7	5.6 ± 0.3	3.9 ± 0.2
Weight (g)	34.3 ± 4.3	36.8 ± 2.8	33.0 ± 2.8	35.7 ± 2.8	32.8 ± 3.7	35.5 ± 3.7	32.1 ± 1.5	33.1 ± 0.9
Urine volume (mL/day)	1.5 ± 0.2	1.2 ± 0.1	1.1 ± 0.2	0.9 ± 0.2	3.0 ± 0.2	3.5 ± 0.4	3.4 ± 0.6	2.2 ± 0.2
Urine Na^+^ excretion (*μ*eq/day)	252 ± 31	248 ± 20	33 ± 4	38 ± 7	1819 ± 160	2002 ± 168	446 ± 50	358 ± 39

### Angiotensin‐II infusion

**Figure 5:**
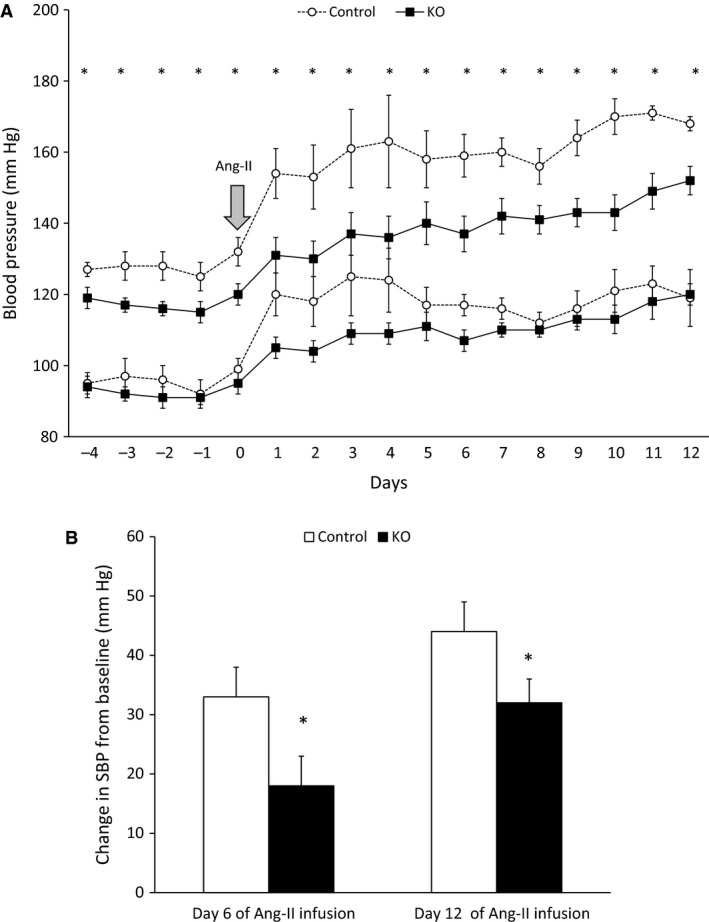
Daily BP in control and AGT KO mice during 14‐day Ang‐II infusion (400 ng/Kg/min). (A) BP was measured via telemetry and mice were fed a normal Na^+^ diet (*N* = 6–8/group). Top two lines show systolic, and bottom two lines show diastolic BP; (B) Change in systolic BP from baseline, following Ang‐II infusion (*N *= 6–8 group). *P < 0.05 versus control mice.

Ang‐II infusion at 400 ng/kg/min increased systolic BP in control mice starting on day 2 and remained elevated throughout the study period (Fig. 5). In contrast, AGT KO mice had an attenuated hypertensive response throughout the infusion. Similar trends were observed in diastolic BP although significant differences between the two groups were noted only on days 2–4 (Fig. 5). Food intake, water intake, body weight, urine volume, and urine Na^+^ excretion were similar between the two groups post‐Ang‐II infusion (Table 1).

### Renin–angiotensin system parameters

**Figure 6:**
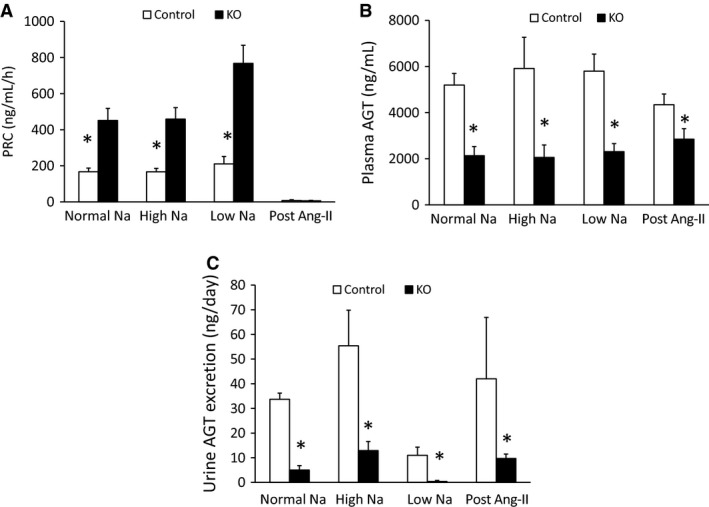
Renin–angiotensin system parameters in control and AGT KO mice. (A) Plasma renin concentration; (B) plasma AGT; and (C) urine AGT levels on normal, low and high Na^+^ diets and following 14 day Ang‐II infusion (*N* = 8–10/group). *P < 0.05 versus control mice.

Before Ang‐II infusion, PRC was elevated in the AGT KO mice on all three Na^+^ diets (Fig. 6A). Following Ang‐II infusion, both KO and control mice had similarly and markedly suppressed PRC levels. Plasma AGT levels were reduced in AGT KO mice by approximately 50% regardless of Na^+^ intake (Fig. 6B) and remained unchanged following Ang‐II infusion. Urinary AGT excretion was markedly reduced in AGT KO mice before and following Ang‐II infusion (Fig. 6C). Low Na^+^ intake reduced urinary AGT excretion in both groups, although the AGT KO mice had markedly decreased urinary AGT levels compared to controls. In contrast, high Na^+^ diet increased urinary AGT excretion in both groups but with fourfold higher levels in control compared to KO mice (Fig. 6C).

### Renal Na^+^ transporter expression

**Figure 7:**
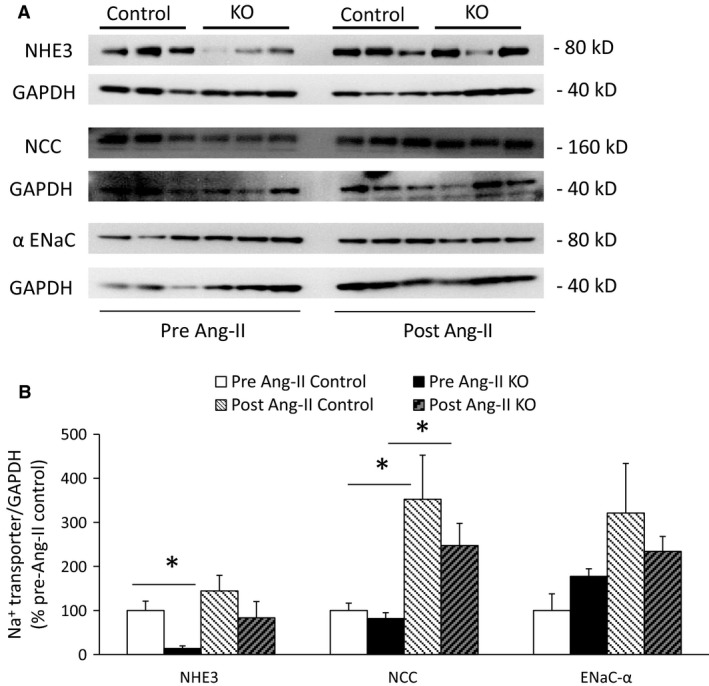
Renal Na^+^ transporter expression in control and AGT KO mice on a normal Na^+^ diet and following 14‐day Ang‐II infusion. (A) Western blots of whole kidney lysates; (B) Densitometry of Na^+^ transporter expression adjusted to GAPDH and represented as % pre Ang‐II control (*N* = 3/group). *P < 0.05 versus control mice.

Before Ang‐II infusion, compared to control mice, AGT KO mice had 85% lower expression of Na^+^/H^+^ exchanger (NHE3) (Fig. 7), but similar expression of Na^+^/Cl^−^ co‐transporter (NCC) and epithelial Na^+^ channel (ENaC)‐*α* subunit (Fig. 7). Ang‐II infusion increased NCC expression (Fig. 7); NCC expression was lower in AGT KO mice post‐Ang‐II infusion compared to controls. Although NHE3 and ENaC‐*α* expression tended to increase in both groups following Ang‐II infusion, this did not achieve statistical significance. Furthermore, no detectable differences in NHE3 or ENaC‐*α* were observed control and AGT KO mice post‐Ang‐II infusion.

### Urinary albumin excretion

**Figure 8:**
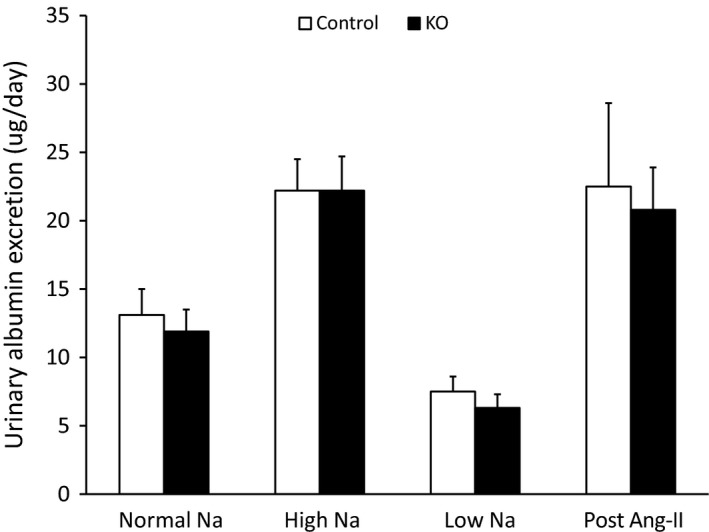
Urine albumin excretion on normal, low, and high Na^+^ diets and following 14 day Ang‐II infusion (*N* = 8–10/group).

No differences were observed in urinary albumin excretion between control and AGT KO mice under all three diets and following Ang‐II infusion (Fig. 8).

## Discussion

This study addressed two key questions about nephron‐derived AGT: (1) the functional significance of nephron AGT in BP regulation; and (2) whether urinary AGT excretion reflects tubular AGT synthesis. Compared to controls, nephron AGT KO mice demonstrated a marked reduction in BP with low Na^+^ intake and following Ang‐II infusion. However, plasma AGT levels were reduced by about 50% in the AGT KO mice relative to controls, indicative of partial deletion of systemic AGT. Notably, PRC was increased in AGT KO mice across all Na^+^ diets, likely due to a compensatory systemic RAS response to lower circulating plasma AGT levels. Consequently, it is not easy to delineate if the decreased BP observed under normal variations in dietary salt in AGT KO mice is due to reduced systemic AGT levels and/or to absence of nephron‐derived AGT. One way to address this issue would be to infuse systemic AGT into AGT KO mice so that plasma AGT levels are restored to normal levels. However, in preliminary studies not described in this work, we were unable to substantially raise systemic AGT levels in AGT KO mice despite continued subcutaneous administration of large doses of AGT. Another possibility is to perform kidney cross‐transplantation studies to examine whether transplanting an AGT KO mouse kidney into a control mouse would reduce BP (Crowley et al. 2005). Such studies are technically challenging and would require multigenerational breeding to change the current genetic background from C57BL/6J to 129Sv strain (since C57BL/6J typically have two renal arteries per kidney, making transplantation problematic). Nevertheless, the attenuated hypertensive response to Ang‐II infusion in the AGT KO mice, wherein PRC was suppressed to similar degree as controls, suggests that nephron‐derived AGT might contribute to BP regulation, at least under elevated circulating Ang‐II levels.

Urinary AGT excretion was markedly lower in the AGT KO mice compared to controls both prior to and following Ang‐II infusion. These results are in agreement with findings wherein systemically infused human AGT into rats was detected in negligible amounts in the urine (Kobori et al. 2003). In contrast, a previous study reported that PT‐specific AGT KO mice only had a 50% reduction in urinary AGT levels, whereas liver‐specific AGT KO mice had no change in urinary AGT excretion (Matsusaka et al. 2012). A possible explanation for the only moderately reduced urine AGT in the PT‐specific AGT KO studies is incomplete ablation of nephron AGT synthesis. The PT‐specific AGT KO mice had renal AGT levels that were not substantially affected, whereas we found that renal AGT levels were markedly lower in nephron AGT KO animals. While it is realized that systemic AGT levels were also reduced in our AGT KO mice, they were still about 50% of control levels and could not fully account for the marked reduction in renal AGT content. Taken together, these results support the notion that urinary AGT excretion is primarily derived from the nephron. This finding is particularly important since several clinical studies have demonstrated urinary AGT excretion to be a significant predictor of hypertension and kidney disease (Alge et al. 2013; Michel et al. 2014; Park et al. 2015; Yang et al. 2015).

It is not entirely clear why liver AGT protein remains unaffected in the AGT KO mice, whereas liver AGT mRNA and plasma AGT are reduced compared to control mice. One reason might be that, despite reduced liver AGT mRNA levels, there is posttranslational regulation of AGT protein such that plasma AGT levels are not reduced to the same degree as predicted by the liver AGT mRNA content. It is also possible that liver AGT levels do not accurately reflect AGT synthesis and that relatively small amount of AGT are stored in the liver. Hence, plasma AGT levels may be a better indicator of liver AGT production rather than liver AGT mRNA or protein. Furthermore, sources outside the liver, such as adipose tissue, might contribute to plasma AGT levels (Yiannikouris et al. 2012). However, it should be noted that when liver AGT is completely deleted using a liver‐specific KO model (Matsusaka et al. 2012), AGT mRNA and protein in the liver as well as plasma AGT are virtually undetectable, supporting the notion that the vast majority of plasma AGT derives from the liver.

Nephron‐derived AGT could potentially regulate BP through Na^+^ reabsorption via luminal Ang‐II generation (Navar et al. 2011). Luminal Ang‐II can stimulate NHE3 activity in the PT (Saccomani et al. 1990; Reilly et al. 1995) and ENaC activity in the distal nephron (Peti‐Peterdi et al. 2002; Mamenko et al. 2012). No differences were observed in urinary Na^+^ excretion between AGT KO mice and controls with varying Na^+^ intake and post Ang‐II infusion; this does not mean that such differences did not occur, but that they were below our ability to detect them, possibly due to inherent intermouse variability. However, renal NHE3 expression was lower in the AGT KO mice compared to controls on a normal Na^+^ diet. Following Ang‐II infusion, expression of NHE3, NCC and ENaC‐*α* tended to be lower in the AGT KO mice as compared to controls but did not achieve statistical significance. Notably, Na^+^ transporter activity or membrane abundance were not directly assessed, hence conclusions about changes in transporter activity must await such detailed analysis.

This study has some limitations. Although the primary goal was to develop nephron‐specific AGT deletion, partial targeting of systemic AGT was observed, complicating the interpretation of our findings. To the best of our knowledge, no other Cre‐expressing mouse model is able to completely target the entire nephron or all PT segments. While it is known that Pax8‐rtTA‐based gene deletion can target periportal hepatocytes (Traykova‐Brauch et al. 2008; Mathia et al. 2013), the degree to which a given hepatocyte protein is deleted depends upon the location of the targeted protein within hepatocytes. Thus, the reduction in systemic AGT in our model is likely due, at least in part, to targeting of AGT synthesis sites in the liver. Second, all studies were conducted in male mice hence inherent sex differences in nephron AGT synthesis and function cannot be excluded. Lastly, as described earlier, it is difficult to delineate whether the BP phenotype observed in this study under varying Na^+^ intake results from reduced nephron or systemic AGT levels.

In summary, our findings suggest that nephron‐derived AGT may be partly involved in mediating the hypertensive effects of Ang‐II infusion. In addition, our findings suggest that urinary AGT excretion is primarily derived from the nephron. Whether nephron AGT is involved in BP regulation under physiological conditions remains to be determined.

## Conflict of Interest

None declared.
